# EPID based *in vivo* dosimetry system: clinical experience and results

**DOI:** 10.1120/jacmp.v17i3.6070

**Published:** 2016-05-08

**Authors:** Sofia Celi, Emilie Costa, Claas Wessels, Alejandro Mazal, Alain Fourquet, Pascal Francois

**Affiliations:** ^1^ Medical Physics Department Institut Curie Paris France; ^2^ Medical Physics Department CHU de Poitiers Poitiers France

**Keywords:** *in vivo* dosimetry, EPID, EPIgray, quality assurance

## Abstract

Mandatory in several countries, *in vivo* dosimetry has been recognized as one of the next milestones in radiation oncology. Our department has implemented clinically an EPID based *in vivo* dosimetry system, EPIgray, by DOSISOFT S.A., since 2006. An analysis of the measurements per linac and energy over a two‐year period was performed, which included a more detailed examination per technique and treatment site over a six‐month period. A comparison of the treatment planning system doses and the doses estimated by EPIgray shows a mean of the differences of 1.9% (±5.2%) for the two‐year period. The 3D conformal treatment plans had a mean dose difference of 2.0% (±4.9%), while for intensity‐modulated radiotherapy and volumetric‐modulated arc therapy treatments the mean dose difference was −3.0 (±5.3%) and −2.5 (±5.2%), respectively. In addition, root cause analyses were conducted on the *in vivo* dosimetry measurements of two breast cancer treatment techniques, as well as prostate treatments with intensity‐modulated radiotherapy and volumetric‐modulated arc therapy. During the breast study, the dose differences of breast treatments in supine position were correlated to patient setup and EPID positioning errors. Based on these observations, an automatic image shift correction algorithm is developed by DOSIsoft S.A. The prostate study revealed that beams and arcs with out‐of‐tolerance *in vivo* dosimetry results tend to have more complex modulation and a lower exposure of the points of interest. The statistical studies indicate that *in vivo* dosimetry with EPIgray has been successfully implemented for classical and complex techniques in clinical routine at our institution. The additional breast and prostate studies exhibit the prospects of EPIgray as an easy supplementary quality assurance tool. The validation, the automatization, and the reduction of false‐positive results represent an important step toward adaptive radiotherapy with EPIgray.

PACS number(s): 87.53.Bn, 87.55.Qr, 87.56.Fc, 87.57.uq

## I. INTRODUCTION

Over the last few years the efficacy of radiation oncology treatments improved dramatically. However, due to the increase in technical complexity and dose escalation, the risk of secondary effects also rises. *In vivo* dosimetry (IVD) is now widely recommended to avoid major treatment errors^1^, ^2^, ^3^, ^4^, ^5^, ^6^, ^7^, ^8^ and is even mandatory in several countries.^9^, ^10^ It is the ultimate step to secure the treatment before a significant portion of the total dose is already given.

The widely recognized reference in the IVD practice consists in placing dosimeters, such as diodes, thermoluminescent dosimeters, or metal oxide semiconductor field effect transistors (MOSFETs), on the patient's skin or inside the patient to derive the dose at specific points of interest within the patient.^2^, ^4^, ^7^, ^11^ However, in clinical routine, placing detectors on the patient's skin at the entrance and/or exit surface of the beam is not always feasible and requires additional setup time in the treatment room. Therefore, this method is inadequate for modern treatment techniques such as intensity‐modulated radiotherapy (IMRT) or volumetric‐modulated arc therapy (VMAT). Thus IVD remains often limited to the first fraction of the treatment and is still not implemented in every radiotherapy department. In France, where *in vivo* dosimetry is mandatory, only 68% of the centers monitor the entirety of their treatment plans with IVD.

In this perspective, transit dosimetry using amorphous silicon electronic portal imaging devices (EPID) appears to be an interesting alternative for several practical reasons.^(12)^ Portal IVD is easy to use and requires neither additional setup time nor additional detectors. Also, most field incidences can be directly measured. The properties and adaptation requirements for the use of EPID as dosimeters have been evaluated in the literature.^13^, ^14^, ^15^, ^16^, ^17^, ^18^

In our institute, a transit IVD method has been in development and use since 2006. This software, EPIgray, by DOSIsoft S.A. (Cachan, France), has since been commercialized and installed in over 60 centers worldwide. From June 2012 to July 2013, transit dosimetry has been successively introduced in routine at our center for all treatment sites and techniques.

In addition to its use in patient quality assurance, EPIgray can be easily employed to perform more detailed studies of certain patient cases or techniques over extended periods of time. Since recording MV images during the treatment requires neither extra time nor radiological adjustments, a dosimetric study can even be conducted over the whole course of the treatment if necessary. Such studies can either be for the validation of a technique, the confirmation of the stability of the treatment plan, or the study of anatomical or other changes that lead to dosimetric changes (e.g., systematical errors).

The goal of this article is to share the experience of our center with the EPID‐based IVD system, EPIgray, review the results, and discuss the use of transit dosimetry beyond the classic IVD use.

## II. MATERIALS AND METHODS

### A. Context and workflow

The Radiation Oncology Department of our center is equipped with five Varian linacs (Varian Medical Systems, Palo Alto, CA), of multiple photon energies (4 MV, 6 MV, 10 MV, 15 MV, and 20 MV). Each linac carries an EPID, used for *in vivo* dosimetry, pretreatment quality assurance and, in the absence of a low energy imager, for patient setup. The five EPIDs have different resolutions (type AS500 or AS1000) and mechanical precision (support arm system of type R‐arm or Exact‐arm). In addition, the image acquisition systems (IAS) are of different versions (IAS2 and IAS3), newer version having improved reproducibility and linearity. Two accelerators have a kV imaging system (On Board Imaging System or OBI). These parameters are summarized in Table 1. Some treatment sites are treated of two Tomotherapy units, not discussed in this article.

At the time of the study, the treatments were planned on Eclipse v.10 (Varian Medical Systems), using the AAA calculation algorithm. The treatment techniques include conventional radiotherapy, 3D conformal radiotherapy, IMRT (sliding window) and VMAT (RapidArc). The two main clinical sites treated in our institution are breast cancer and prostate cancer.

EPIgray uses the transmitted signal on the EPID to reconstruct the dose at one or several point(s) of interest within the patient. The dose reconstruction method uses a formalism described previously.^19^, ^20^ EPIgray version 2.0.3 — featuring the VMAT option — was used in the studies presented in this paper.

**Table 1:** Treatment delivery system.

			*Imaging system*				
	*Linac*	*Energy (MV)*	*MV*	*kV*	*Set Up*	*Main Techniques*	*Main Treatment Sites*
1	TrueBeam v. 1	6, 10, 15	AS1000 IAS3 E‐arm	OBI	2D kV‐kV	IMRT VMAT	Prostate Head Gynecology
2	Clinac 2300EX	6, 20	AS500 IAS2 R‐arm		2D MV‐MV	3D CRT FiF	Breast Metastasis
3	Clinac 2100 C/S	4, 10	AS500 IAS2 R‐arm		2D MV‐MV	3D CRT FiF	Breast Metastasis
4	Clinac 2100 C/S	6, 20	AS500‐II IAS3 E‐arm	OBI	2D kV‐kV	IMRT VMAT	Pediatrics Prostate
5	Clinac 600	6	AS1000 IAS3 E‐arm		2D MV‐ MV	3D CRT FiF	Breast Head Metastasis

The portal imagers are calibrated to the reference dose following the manufacturer's procedures. The calibration is verified on a weekly basis and a new calibration is performed when the dose response difference is greater than 2%. Two types of images can be recorded by these EPIDs for IVD purposes: integrated dose images used for conformal and IMRT beams, and “cine mode” images used solely for dynamic rotational treatments.

Per monitored fraction, EPIgray determines the dose per beam and interest point in the patient, as well as the total dose per plan and interest point. Each dose difference per fraction/beam/interest point is called an IVD control. As such, a five‐beam plan carrying two interest points results in 10 controls per fraction.

In our center, the points of interest are created during the treatment planning and are not explicitly intended for IVD. The majority of these points are either ICRU reference points in the case of 3D CRT or the isocenter in the case of complex treatments. Additional points may be created, for reference or *in vivo* dosimetry, if needed. Several points are of course placed in the case of treatments with multiple plans.

EPIgray has been previously tested and validated on phantom geometries simulating as close as possible our clinical conditions.^(21)^ A per default tolerance level of 6.5% was established and the system's ability to operate within the specified limits was evaluated. When sufficient data were available to perform a statistical study, this tolerance level was reevaluated and individualized per treatment site and technique. The default tolerance is now of 7.5%, while the tolerance levels per treatment site evolve in a range of (6.5% to 10.5%).

**Figure 1:**
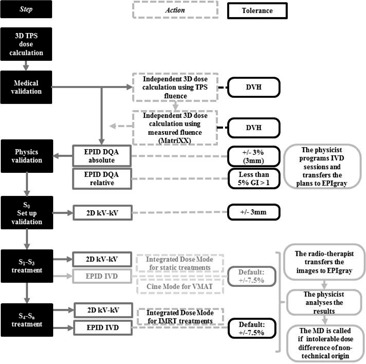
Chart of the workflow of the IVD controls, on the example of IMRT plans.

In our center, IVD was performed for three consecutive fractions at the beginning of every new treatment plan. In Fig. 1 an example of the detailed workflow for IMRT plans is described. If measurement results are above the established tolerance levels, an assignable cause is sought. A consecutive action chain depending on the origin of the IVD problem has been previously established. The problem sources and consecutive actions are detailed in Table 2. If no benign cause can be assigned or the cause has dosimetric consequences, the radiation oncologist is notified and a specific corrective action is adopted.

Due to EPID sensitivity, relative dose difference controls with predicted doses underneath 10 cGy were disregarded. In addition, out‐of‐tolerance results caused by EPID technical faults were eliminated and new IVD images were programmed.

Between 2012 and 2013, most treatment sites and techniques (conformal (3D CRT), Field in Field (FiF), and IMRT) were included in the IVD process. VMAT was added in July 2013. Up to now, the only restriction remains the treatment with noncoplanar beams — where a couch rotation is necessary — because of the risk of collision between the EPID and the couch.

**Table 2:** Observed dose difference causes and established course of action.

*Type of Problem*	*Problem*	*Solution*
Patient deform ation & set up	Anatomical variation (weight, oedema, gas, etc..…)	Verify SSD, thicknesses and replan plan with new CT data if necessary
	Unexpected object in the beam Patient setup	Review patient setup and replan acquisition
Treatment plan & choice of points of interest	Point in low‐dose area
	Find new point of interest and recalculate transit dose
		Point in high‐dose gradient
		Point in high‐dose gradient
		Point close to tongue‐and‐groove area
Point close to an heterogeneity interface	
Machine & EPID	Acquisition interrupted before end of beam irradiation	Replan acquisition
	EPID in wrong position	Calibrate EPID positioning and replan acquisition
	Drift of EPID calibration in dose or machine output	Proceed to a new calibration

### B. Analysis of the clinical IVD results

The main objective of IVD is the verification of an individual treatment fraction in search of errors. The unsatisfying results of a single fraction of a treatment plan are analyzed in the context of the technique, treatment site, and individual patient plan.

#### B.1 Analysis per linac and technique

The configuration of our treatment delivery system is presented in Table 1. As explained above, “integrated dose” images are acquired in the case of static techniques (3D CRT or FiF) and IMRT, beam by beam. Images in “cine” mode are acquired in the case of VMAT treatments, where the irradiation is continuous over the whole arc. The cine mode images of the linac 1, a TrueBeam, are in a new format. This format is incompatible with the treatment delivery system and EPIgray version available to our center. The VMAT plans of linac 1 could thus not been monitored with EPIgray at the time of this study.

To ensure a correct follow‐up, adapt the tolerance limits to the reality of the clinic, and timely detect any systematic error, series of statistical studies are periodically conducted, using the statistical tools embedded in EPIgray, as well as additional in‐house software (Microsoft Excel 2010 and MATLAB v.2.014b (MathWorks, Natick, MA)). These data include mean, median, and standard deviations, frequency distributions, and monitoring of the temporal evolution. The analyses are performed for all patients and for each linac separately and include the dose difference for each individual point of interest per beam per fraction, the dose difference per point of interest per fraction for the whole treatment plan, or the dose difference per point of interest per beam for all fractions combined.

The present analysis was conducted over two years (July 2013 to June 2015). It includes all monitored treatment plans of linacs 2, 3, 4, and 5, and the static (conformal and IMRT) beams of linac 1. This represents 9163 checked beams, for a total of 3163 patients. The study includes the annual statistics and temporal evolution, the results for the individual linacs, and observations made based on the conducted analyses.

#### B.2 Analysis per treatment site

An additional statistical study was undertaken on a subset of the above sample to assert the previously defined tolerances and confront the results for different techniques and treatment sites. All analyzed plans from July 2013 to January 2014 were categorized according to the anatomical treatment site: head, neck and oral (HNO), ophthalmology, lungs, breast, axillary, pelvis, and limbs.

Lymph nodes and metastasis treatment plans were classic, 3D conformal (3D CRT) and were mostly performed on linacs 2, 3, and 5, as presented in Table 1. Two conformal breast treatment methods were in use at our center: treatment in supine position and treatment in lateral position. These were mostly carried out on linacs 2, 3, and 5. Head treatments were planned using the IMRT technique on linacs 1, 4, or 5. Pelvis treatments were either VMAT or IMRT, performed on linacs 1 and 4. Different ophthalmologic, neck/oral, and thorax treatments were carried out with all available techniques, 3D CRT, IMRT or VMAT, on linacs 1, 4, and 5.

This subset includes 857 patients corresponding to 2837 checked beams, 2444 treatment fractions, with an average of 2 points of interest per patient. The analysis includes statistical data as cited above, mean, median, and standard deviations (SDs), as well as a comparison to the previously defined tolerances and an estimation of the number of failing beams.

### C. Root cause analyses

In addition to the statistical analyses above, two technical studies were performed with the IVD system to investigate the root causes of the observed differences.

#### C.1 Conformal technique: breast cancer cases

**Figure 2:**
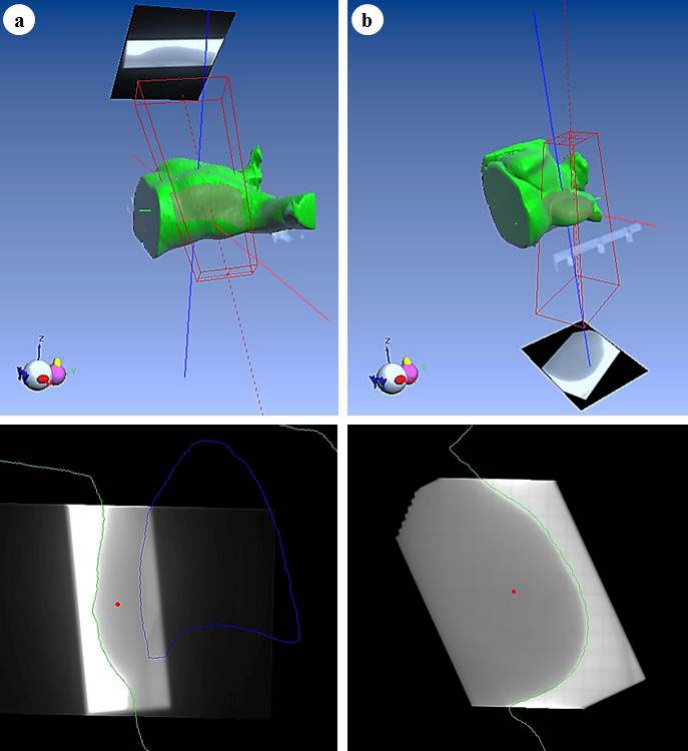
Example of IVD images and points of interest of breast treatment plans in (a) supine and (b) lateral positions in EPIgray.

Breast cases represent about 50% of the plans monitored with IVD. IVD images were recorded during the 2nd to 4th fractions, the 1st fraction being restricted to patient positioning. Two treatment techniques are possible: in lateral^(22)^ (isocentric lateral decubitus [ILD]) or in supine position, as illustrated in Fig. 2. The treatment in the supine position is a conventional technique. The beams are tangential and may include subfields. In the case of an ILD treatment, the patient is placed on the side. The beams are opposite and wedges may be used. The choice of technique is based on several criteria such as the morphology of the patient, the volume of the treated breast, and the advancement of the disease. The treatment of the axillary, internal mammary lymph nodes, and boosts are planned separately.

During the routine use of EPIgray, it was noted that IVD results of breast treatments were worse than expected and that integrated dose images of breast treatment beams were subjectively often misaligned with the TPS patient contours.

A root cause analysis was thus undertaken on the IVD measurements of 176 supine and 148 lateral breast treatment plans to study the influence of these misalignments on the IVD results and to establish the quantity and origin of the observed shifts.

#### C.2 Static IMRT and dynamic arc therapy: prostate cancer cases

**Figure 3:**
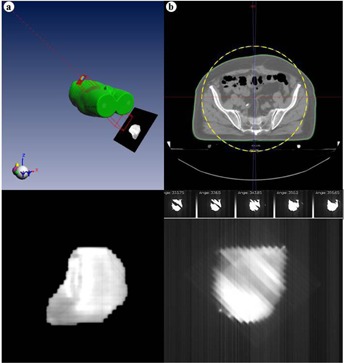
Example of IVD images of a prostate treatment obtained for (a) an IMRT beam and (b) a VMAT arc, in EPIgray.

For IMRT and dynamic arc therapy, transit dosimetry represents an important progress in comparison with classic IVD, where the measurement for such small and modulated fields at different angles is technically difficult. These techniques and IVD images for a beam and an arc angle are illustrated in Fig. 3.

About 160 prostate cancer patients are treated per year at our center. Until 2013, all these patients were treated with IMRT and most are treated with RapidArc since. The IMRT plans are calculated with five beams of energy 20 MV for a total dose of either 76 or 80 Gy. The prostate treatment with VMAT is carried out in two plans of energy 6 MV, pelvis and prostate, each carrying two arcs.

In the case of IMRT, all plans are monitored with IVD. Setup was performed on the first three days of the treatment. Then IVD images were usually taken during the 4th to 6th fractions. Since June 2013, the RapidArc plans on one of the linacs are monitored with IVD. For VMAT, where setup imaging is done daily, IVD images are taken on the first 3 fractions. Relative dose differences must often be disregarded for fields underneath 10 cGy, where absolute dose difference is preferred. The main difficulty of these IVD controls is the lack of visual support. Due to the small size of the fields, when the tolerance value is exceeded, the image does not provide any additional information, as it does in the case of larger fields. Instead, one relies on experience with such difficult fields and additional sources of information to find an explanation for the failing beams and arcs.

To verify the assumptions made about the failing complex fields, the results of the IVD controls of 20 IMRT prostate plans of 2012 and 14 VMAT prostate plans of 2014‐15 were confronted to the following plan parameters, determined from the DICOM plan using an in‐house MATLAB v.2.014b software.

1. The modulation complexity, described by the Modulation Complexity Score (MCS),^23^, ^24^ for IMRT and VMAT:
  (1) $$MCS_{beam}=\sum_{i=1}^{Z-1}\left(\frac{LSV_{CP_{i}+}LSV_{CP_{i+1}}}{2},\frac{AAV_{CP_{i}+}AAV_{CP_{i+1}}}{2},\frac{MU_{i,i+1}}{MU_{beam}}\right)$$
  with the Leaf Sequence Variability (LSV) per control point $CP_{i}$, for a total number Z of control points:
  (2) $$LSV_{CP}={\left[\frac{\sum_{n}^{Z-1}(pos_{\text{max}}-\left|pos_{n}-pos_{n+1}\right|)}{(N-1)\times pos_{\text{max}}}\right]}_{left}\cdot {\left[\frac{\sum_{n}^{Z-1}(pos_{\text{max}}-\left|pos_{n}-pos_{n+1}\right|)}{(N-1)\times pos_{\text{max}}}\right]}_{right}$$
  where $pos_{n}$ is the position of the left/right leaf n and $pos_{max}$ is the maximal position variability of the left/right leaf bank at control point $CP_{i}$, for a total number N of open leaves.
  The Aperture Area Variability (AAV) per control point is given by:
  (3) $$AAV_{CP}=\frac{\sum_{n=1}^{N}\left(\{pos_{n,left}\}-\{pos_{n,right}\}\right)}{N\times (\text{max}\{pos_{n,left}\}-\text{max}\{pos_{n,right}\})}$$
2. The position of the point of interest in the field or under the leaves, given by the point position index (PI):
  (4) $$PI_{beam}=\sum_{i=1}^{Z}PI_{CP_{i}}\cdot BM_{CP}\;\text{With}\;PI_{CP_{i=}}\{\begin{array}{cc} 1 & if\;po\text{int}\;in\;field\\ 0 & if\;po\text{int}under\;the\;leaves \end{array}$$
  where $BM_{CP}$ is the beam meterset.
3. The variability of the dose rate (Dr) during an arc in the case of VMAT:
  (5) $$Dr_{arc}=\frac{\sum_{i}^{Z-1}\left|Dr_{CP_{i}-}Dr_{CP_{i}+1}\right|}{(Z-1)\times {\left|Dr_{CP_{i}-}Dr_{CP_{i}+1}\right|}_{\text{max}}}$$
  with $DR_{CPi}$ being the dose rate at control point.

The IVD results are given in terms of mean and standard deviation of the dose difference per beam and per point of interest averaged over all monitored fractions, each plan containing an average of two points of interest.

## III. RESULTS & DISCUSSION

### A. Statistical analysis per linac and technique

**Figure 4:**
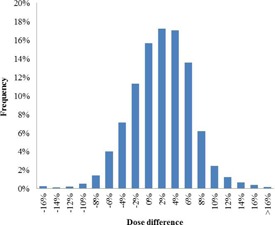
Dose difference distribution of the IVD results for the period of July 2013 to June 2015.

For the two‐year period (July 2013 to June 2015), the mean of the differences between the TPS dose and the dose estimated by EPIgray per control was 1.9% (±5.2%), with an average of 1.9 points of interest per plan and 2.9 beams per plan. The mean of dose differences averaged over all monitored fractions for each beam and point of interest is similar: 1.7% (±4.6%). The dose difference distribution is illustrated in Fig. 4. The normal distribution of the data is confirmed by the Kolmogorov‐Smirnov Test and testifies of a globally controlled process.^(25)^

On average, the 1st, 2nd, and 3rd fractions have very similar results: 1.8% (±5.1%) for the 1st monitored fraction, 2.0% (±5.2%) for the 2nd, and 2.0% (±5.4%) for the 3rd, per point of interest, per beam.

The mean and standard deviation of the calculated dose differences per point of interest, per beam, per fraction were calculated for the linacs and techniques, and are presented in 3, 4. Discrepancies are observed between the different techniques (Table 4), but little difference is seen between different energies of the same linac for the same technique (Table 3).

**Table 3:** Results of the analysis per linac over a two‐year period (July 2013 to June 2015).

		*Dose Differences Per Point of Interest, Per Fraction, Per Beam*			*Dose Differences Averaged Over All Monitored Fractions Per Beam, Per Point of Interest*		
		*Patients*	*Number*	*Mean*	*SD*	*Number*	*Mean*	*SD*
LINAC 1	6 MV	95	670	−1.5%	5.0%	292	−1.4%	4.3%
	10 MV	41	261	1.2%	4.6%	105	1.1%	4.4%
	15 MV	139	906	−0.9%	3.8%	378	−0.5%	4.0%
LINAC 2	6 MV	606	7624	1.6%	4.7%	2766	1.4%	4.0%
	20 MV	158	875	2.4%	5.2%	358	2.4%	4.4%
LINAC 3	4 MV	571	8449	1.4%	5.1%	2977	0.9%	4.8%
	10 MV	194	1444	1.8%	3.8%	551	1.6%	3.7%
LINAC 4	6 MV	384	3642	0.3%	4.8%	1438	0.1%	4.3%
	20 MV	225	1333	2.6%	4.6%	572	2.5%	4.4%
LINAC 5	6 MV	750	8903	3.9%	5.6%	3124	3.8%	4.4%
TOTAL		3163	34107	1.9%	5.2%	12561	1.7%	4.6%

**Table 4:** Results of the analysis per technique over a two‐year period (July 2013 to June 2015).

*Technique*	*Patients Number*	*Controls Number*	*Beams Number*	*Mean Dose Difference*	*SD*
CONFORMAL	3080	33136	7730	2.0%	4.9%
IMRT	27	383	75	−3.0%	5.3%
VMAT	56	582	107	−2.5%	5.2%
TOTAL	3163	34107	7912	1.9%	5.2%

The plans of linac 2 have a mean dose difference per beam averaged over all fractions of 2.4% (±5.2%) for 20 MV plans and 1.6% (±4.7%) for 6 MV plans. Likewise, the 4 MV beam of linac 3 has a mean dose difference value of 1.4% (±5.2%) and the 10 MV plans have a slightly higher mean dose difference of 1.8% (±3.8%). All plans are static. The static plans of linac 4 show comparable results: 2.6% (±4.6%) for 20 MV and 1.0% (−4.4 %) for 6 MV. On the other hand, the mean dose differences for the complex plans are larger: −2.5% (±5.2%) for VMAT plans and −2.7% (±5.7%) for IMRT plans. The three energies of linac 1 have results similar to the above: 1.3% (±4.6%) for 10 MV, −0.6% (±3.8%) for 15 MV, −1.5% (±5.0%) for 6 MV and larger dose differences for IMRT plans than static plans. The higher results seen for the linac 5 reflect a systematic error due to the configuration of the software for this linac.

### B. Statistical analysis per site and technique

**Figure 5:**
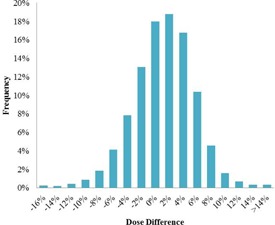
Dose difference distribution of the IVD results for the period of July 2013 to January 2014.

The mean dose difference between the predicted and reconstructed dose for the studied six‐month period is 1.2% (±4.5%). 7.4% of the controlled beams exceed the default tolerance of 7.5%. The distribution of the dose differences over the six‐month time period is shown in Fig. 5.

The results of analyses for all sites and techniques are detailed in Table 5.

Conventional techniques score 1.4% (±4.1%) in average. It can be observed that among conventional techniques, the treatments of the breast in supine position score worse than other sites treated with a similar technique. The best results are obtained for treatment plans of axillary and breast in lateral position. For the IMRT and VMAT techniques, the mean dose difference is higher than for 3D CRT plans, −2.2% (±4.5%) for VMAT and −2.8% (±5.1%) for IMRT plans. The worst results with VMAT are obtained for the neck and oral treatments (−4.3%, ±4.1%), and with IMRT the worst results were obtained for a thorax plan. These reflect the difficulties of the treatment site — high heterogeneity and complex setup — as well as the complexity of the used techniques, such as the modulation of the beams. The best results are observed for pelvic plans (−0.4%±5.5% with VMAT and −2.6%±4.2% with IMRT). The large standard deviations of the internal mammary lymph nodes are due to the position of the reference points for this treatment site. In the case of a slight setup error, the water‐equivalent thickness crossed by the beam varies greatly. As a result, the EPID signal varies and an important dose difference is calculated.

**Table 5:** Statistical analysis per treatment site for a six‐month period (June 2013 to January 2014).

*Site*	*Controls Number*	*Beams Number*	*Mean Dose Difference*	*SD*	*Tolerance Levels*
Breast ‐ supine	3882	1342	2.2%	4.5 %	7.5%
Axillary	2804	967	0.5%	4.9%	7.5%
Metastasis	2630	991	1.8%	3.9%	7.1%
Breast ‐ lateral	2508	833	0.8%	3.1%	6.7%
Internal mammary lymph nodes	401	140	1.9%	8.4%	10.0%
Limb	192	85	−1.2%	5.0%	8.2%
Thorax	189	62	−1.1%	6.3%	7.5%
Ophthalmology	94	38	0.1%	5.0%	8.7%
Neck & Oral	145	51	−2.2%	5.4%	7.5%
Pelvis	125	30	−1.8%	4.9%	8.0%
Head	65	17	−1.8%	5.4%	7.5%

The statistical results lay within the previously determined tolerances. These results mirror the clinical problems often encountered for each type of technique or location. As an example, the results for thorax treatment and neck/oral cases reproduce the difficulties of heterogenic regions. On the other hand, technical problems, such as EPID faults, variability, and calibration difficulties, influence the results of the conducted measurements and may create the illusion of a false‐positive, or worse, a false‐negative, IVD control. Our experience concords with the results published for other transit dosimetry systems.^26^, ^27^

### C. Root cause analyses

#### C.1 Breast cases

During the statistical analysis, it was confirmed that the lateral breast treatments generally result in better dosimetric conformance than the supine breast treatment technique. Moreover, the acceptable statistical data for breast treatments in supine position cited above are only obtained after manual corrections of the alignment of the images of supine treatments.

In fact, for a sample of 176 breast/supine plans, a misalignment was observed in 38% of the checked beams. By contrast, the number of misaligned lateral breast treatment images was insignificant: less than 2% of the beams for a sample of 148 breast/lateral plans.

**Figure 6:**
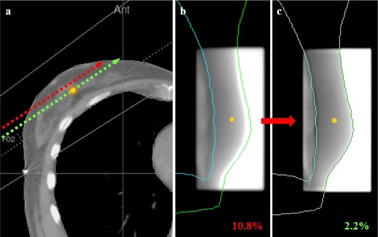
Illustration of the effect of positioning errors on IVD results: (a) in presence of a setup error, the point of interest receives the correct dose but is associated with the wrong CT parameters, such as patient thickness; (b) the signal at the reference point is different than expected, thus a large dose difference is calculated; (c) by realigning the image with the TPS contours, the correct signal is reassigned to the reference point.

While an offset of the image may reflect a patient positioning error or mechanical fault, this is not a dosimetric error; the point of interest received the right dose, but the corresponding signal on the EPID is associated to the wrong plan parameters (position and thickness on the CT). In absence of other error causes than the image offset, a manual realignment of the image was thus performed. This is schematically illustrated in Fig. 6.

Before this correction, 21% of controls of breast/supine treatments were above the tolerance established for this site (mean dose difference of 4.1% (±4.9%)). After the correction, only 5.7% were above the tolerance level (mean dose difference of 2.7% (±3%)).

The performed manual correction of the image position has two components: the lateral (X) and longitudinal (Y) directions of the EPID. The average manual correction in the X direction is −0.5 mm (±6.4 mm), while the average in the Y direction is −3.1 mm (±21 mm).

**Figure 7:**
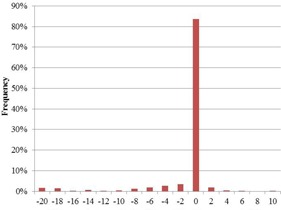
Distribution of the differences between recorded EPID shifts and the Y component of the manual translation performed on IVD images.

First, the positioning of the EPID was investigated. In some cases, the EPID may be voluntarily shifted from its standard position if required by the plan configuration or to avoid collision. In others, the EPID may have been mispositioned. By confronting the manual realignment of the image and the recorded EPID shifts, it was noted that 90% of the manual corrections of the image position in the longitudinal (Y) direction were within ±2 mm of the recorded EPID shifts in that direction (see Fig. 7). The image shifts in the Y direction are thus due to offsets of the EPID in the longitudinal direction. Based on these results, a correction of the position of the image in accordance with the recorded deviation of the imager was developed for EPIgray.

**Figure 8:**
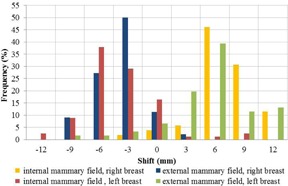
Distribution of the transit image translations in the imager's lateral (X) direction, for internal and external mammary fields.

No correlation between EPID shifts and manual translations in the X direction were found. However, the manual realignment of the image in the lateral (X) direction could be fitted into distinctly separate distributions, depending on the incidence of the tangential beam and the treated side (see Fig. 8). According to the observed distributions, the image misalignments in the X direction tend away from the patient — the patient positioning error seems to derive from the effort of radiographers to spare the lung.

These observations create awareness for this positioning issue that would have otherwise stayed unknown and may become important if other techniques are applied. DOSIsoft S.A. has picked up this problem and a new automatic setup correction algorithm is being developed for EPIgray.^(28)^

#### C.2 Prostate cancer cases

The 20 IMRT prostate plans resulted in a mean dose difference of 1.2% (±3.4%), for a total number of 97 monitored beams and 2 points of interest per plan in average. The 14 VMAT plans resulted in 2.8% (±5.9%), for a total number of 28 arcs (14 for pelvic plans and 14 for prostate plans) and 2 points of interest per plan in average. The mean dose differences of both techniques are within the expected range, despite expected difficulties such as the positioning of the point of interest, modulation complexity of the plans, and low monitor units per image. The results and parameters for the different IMRT and VMAT beams are presented in Table 6.

The mean MCS of the IMRT prostate plan beams is 0.32 (±0.07) and the mean MCS for the VMAT prostate arcs is 0.26 (±0.07), matching the results encountered in the literature.^(29)^ According to the calculated point index (PI), the points of interest are in the field more than 50% of the time only for 23% of the IMRT beams and 32% of the VMAT arcs. The dose rate variability of the VMAT prostate plans is 22%, on average, for pelvis plans and 13% for prostate plans.

The main points of interest (isocenters) of the IMRT plans have a mean dose difference of 0.2%, lower than the mean for other points (2.2%). However all points of interest present similar MCS and PI averages.

The primary arc of the VMAT plans, the arc with the highest predicted dose, has a lower mean dose difference (1.0%) than the arc with the lowest predicted dose, or secondary arc (3.5%). The primary arc has a slightly lower complexity and higher point indices.

The calculated parameters were confronted to the IVD results, in particular the controls exceeding the tolerances established for pelvic treatments. In the case of IMRT, the beams with a mean dose difference above the established tolerance have in average a higher modulation complexity than those beams in the tolerance range (0.27 vs. 0.33). Similarly, 92% of the points of interest of IMRT beams that exceed the tolerance limits are in the field less than 50% of the treatment time, against 74% of the points of interest for the IMRT beams below the tolerance. All “out of tolerance” beams had a MCS lower than 0.35, while the MCS of beams in the tolerance ranged between 0.19 and 0.5 for IMRT, and 0.15 and 0.43 for VMAT.

Though a direct correlation between the plan parameters and the dose differences calculated during IVD cannot be established, some tendencies can be observed. In particular, beams that are above the tolerance tend to have lower complexity scores, and therefore a higher modulation complexity. The points of interest in such plans tend to be less exposed, leading to lower signal and worse reconstructions from the EPID image. The dose rate variability of the prostate VMAT plans is low and has no influence on the IVD outcome.

**Table 6:** Calculated plan parameters for 97 IMRT beams and 28 VMAT arcs.

	*Beam*	*Number of Beams*	*Mean Predicted Dose (Gy)*	*Mean Dose Difference*	*SD*	*MCS*	*PI*	*DR*
IMRT	Posterior	20	0.40	−0.4%	2.3%	0.37	0.40	0
	Lateral left	20	0.35	1.2%	2.4%	0.28	0.40	
	Anterior left	20	0.37	2.1%	3.0%	0.35	0.45	
	Anterior right	20	0.37	1.0%	1.9%	0.34	0.48	
	Lateral right	20	0.36	1.5%	2.1%	0.28	0.43	
VMAT	Primary Arc	14	1.2	1.6%	3.5%	0.28	0.49	0.21
	Secondary Arc	14	0.7	3.9%	3.1%	0.24	0.27	0.20

## IV. CONCLUSIONS

Clinical IVD controls of the two‐year period resulted in a mean dose difference of 1.9% ±5.2%. The best results were obtained for static (conformal RT) plans (2.0%±4.9%). Both complex, dynamic techniques (IMRT and VMAT) had a comparable mean dose difference (−3.0%±5.3% and −2.5%±5.2%). Our statistical results are satisfactory and in accordance with the recommendations. The distribution of the statistical values speaks for a controlled process. The statistical results per technique and site lie within the empirically established tolerance limits. The obtained results for different sites and techniques reflect the common difficulties encountered in other quality assurance practices: heterogeneity and modulation complexity, for example.

The analysis of the breast treatments revealed the origins of the observed differences between treatment techniques and the shifts of IVD images. Consequently, algorithms for an automatic repositioning of the image are being developed by DOSIsoft S.A. On the other hand, few conclusions could be drawn from the comparison of plan parameters to the IVD results, though a tendency can be seen between the parameters and the IVD results below the tolerance threshold. Additional studies will be undertaken to expand the root cause analyses to other treatment sites, and to study other influence factors such as the dose gradients and heterogeneities.

The day‐to‐day practice of IVD has allowed us to expand its use from the basic traditional patient verification to additional uses such as systematic uncertainties' studies and backup QA. However the use of transit dosimetry in routine still represents an important workload for a major center. It is clear that future system integration and automatization are mandatory for a routine application of IVD. Then, the daily IVD controls will be performed allowing us to detect incidents that, for now, remain unrevealed, and to adapt our planning to the patient's actual needs.

## ACKNOWLEDGMENTS

We would like to thank the physicists, technicians, radio‐oncologists and radiotherapists of our center for their patience and support during the different phases of the development and implementation in clinical routine of the EPIgray *in vivo* dosimetry system. A special thanks to the physicists Laurent Bartolucci and Jean‐Luc Dumas, PhD, for their helpful suggestions. We would also like to thank DOSIsoft S.A. for their financial support and the successful cooperation, and in particular Valerie Rousseau, PhD, for her technical support during the development and implementation in clinical routine. Finally, we would like to thank the reviewers and editors of the *Journal of Applied Clinical Medical Physics* for their constructive criticism and helpful suggestions.

## COPYRIGHT

This work is licensed under a Creative Commons Attribution 4.0 International License.

## Supporting information

Supplementary Material
