# Supplementary figures and images for: EPID based in vivo dosimetry system: clinical experience and results
Source: J Appl Clin Med Phys. 2016 May 8;17(3):262–76. doi: 10.1120/jacmp.v17i3.6070 (PMC5690938; doi:10.1120/jacmp.v17i3.6070)

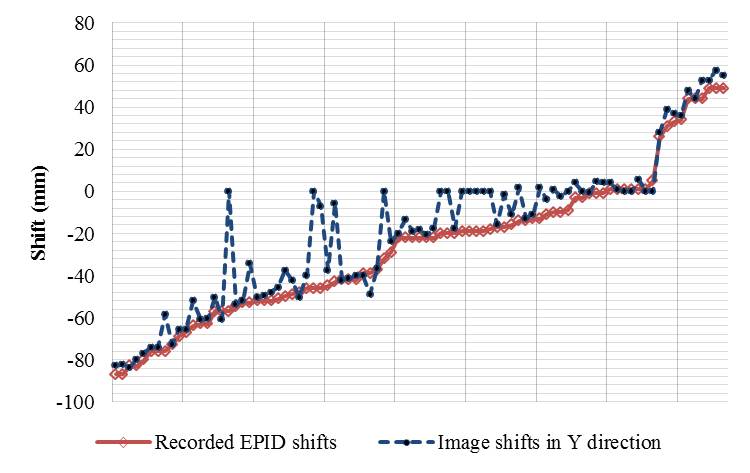

Supplement: Supplementary file 1 — Supplementary Material [file ACM2-17-262-s001.jpg]
